# 1848. Predictors of 6-Month Mortality in *Staphylococcus aureus* Bacteremia: A Population-Based Study in Olmsted County, Minnesota, from 2006 to 2020

**DOI:** 10.1093/ofid/ofac492.1477

**Published:** 2022-12-15

**Authors:** Joya-Rita Hindy, Juan A Quintero-martinez, Brian D Lahr, Daniel C DeSimone, Larry M Baddour

**Affiliations:** Mayo Clinic Rochester, Rochester, Minnesota; Mayo Clinic Rochester, Rochester, Minnesota; Mayo Clinic Rochester, Rochester, Minnesota; Mayo Clinic, Rochester, Minnesota; Mayo Clinic Rochester, Rochester, Minnesota

## Abstract

**Background:**

*Staphylococcus aureus* has high mortality rates, which is influenced by several factors related to the host-pathogen interaction. The current study aims to provide a contemporary evaluation of predictors of 6-month mortality rate in a population-based cohort of incident *Staphylococcus aureus* bacteremia (SAB) cases.

**Methods:**

Retrospective population-based study of 541 adult residents of Olmsted County, MN with monomicrobial SAB from January 1, 2006 through December 31, 2020. The study’s primary outcome was 6-month mortality rate, and a survival analysis was prepared by the Kaplan-Meier method. Multivariable Cox regression was used to investigate risk factors associated with 6-month mortality.

**Results:**

The median (IQR) age of 541 patients with SAB was 66.8 (54.4-78.5) years and 39.6% were female. The median Charlson Comorbidity Index (CCI) was 2 (1-4). SAB cases were healthcare-associated in 49.2% and 56.2% were methicillin-susceptible. Twenty percent of SAB cases had an unknown infection source and 38.3% had complicated bacteremia. There were 7 relapses within the first 12 weeks of follow-up, corresponding to a cumulative incidence rate of 1.3%. There were no re-infections through the remainder of the 6-month follow-up, although there were 2 recurrences after 7 months. Eighty-nine (16.5%) patients died within 1 month of SAB diagnosis and 144 (26.7%) patients died during the 6-month follow-up period. In a multivariable analysis, older age, elevated CCI, unknown source of SAB, and intensive care unit (ICU) admission were significant predictors of an increased 6-month mortality rate. In contrast, presence of diabetes mellitus was significantly associated with a decreased 6-month mortality rate; also, undergoing infectious diseases consultation was associated with reduced mortality in the first 2 weeks, but not for the remainder of the 6-month follow-up period.

**Figure:**
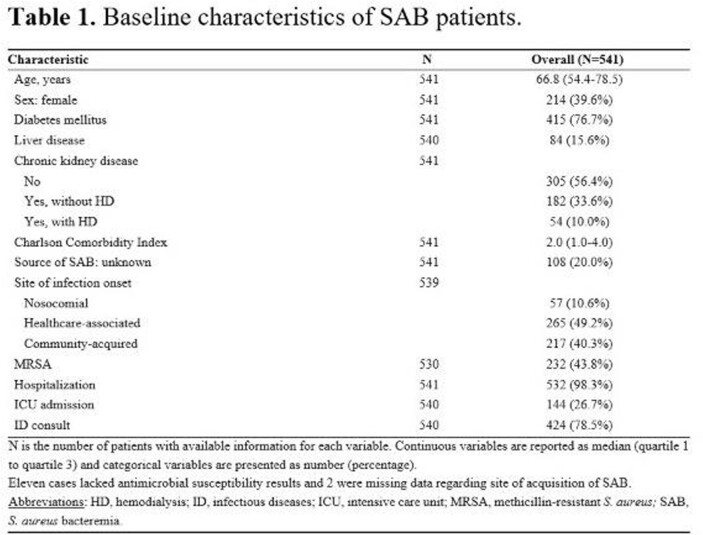

**Figure:**
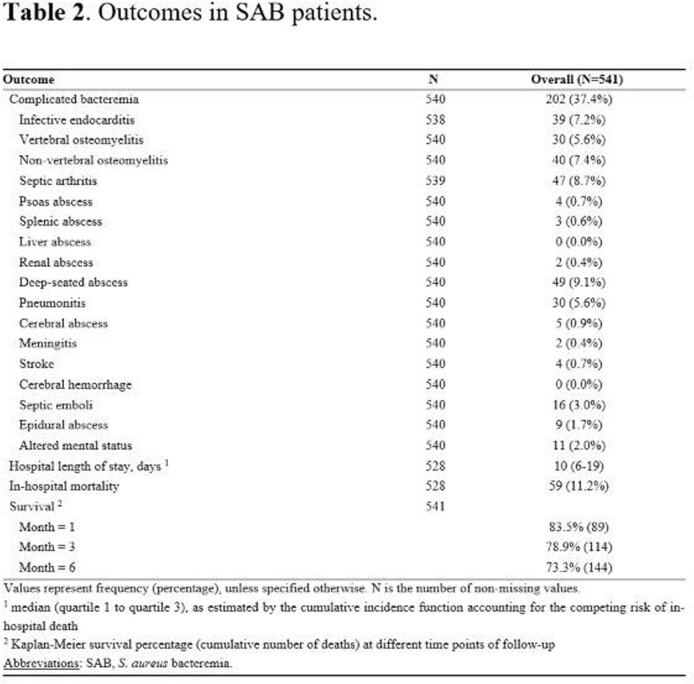

**Figure:**
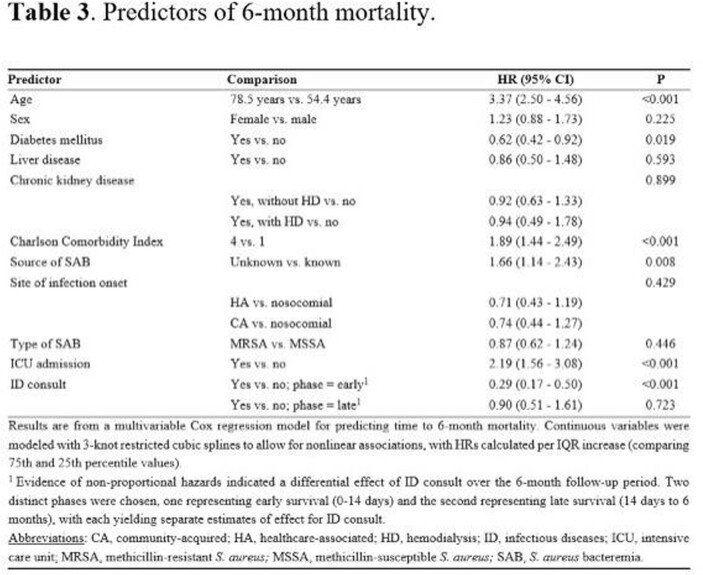

**Conclusion:**

To our knowledge, the current investigation represents the only contemporary population-based study determining significant predictors of mortality in SAB patients, namely, older age, higher CCI, unknown source of SAB, and ICU admission. This study also described one of the lowest 30-day mortality rates reported over the past 2 decades.

**Figure 1.**
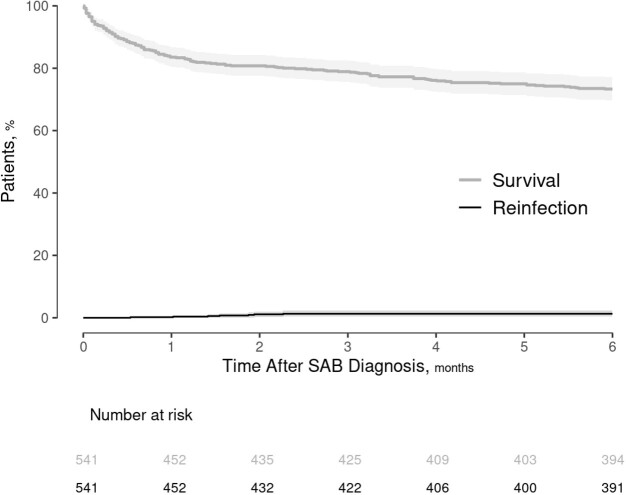
Survival and reinfection rates of patients with SAB over 6 months.

Abbreviations: SAB, Staphylococcus aureus bacteremia.

Foot notes: The overall survival curve was derived by the Kaplan-Meier estimator, while the curve for reinfection was estimated by the cumulative incidence function taking into account the competing risk of death.

**Disclosures:**

**Larry M. Baddour, M.D.**, Boston Scientific: Advisor/Consultant|Botanix Pharmaceuticals: Advisor/Consultant|Roivant Sciences: Advisor/Consultant|UpToDate, Inc.: Royalty payments - authorship duties.

